# Co-expression-wide association studies link genetically regulated interactions with complex traits

**DOI:** 10.1101/2024.10.02.24314813

**Published:** 2024-12-13

**Authors:** Mykhaylo M. Malakhov, Wei Pan

**Affiliations:** 1 Division of Biostatistics and Health Data Science, School of Public Health, University of Minnesota, Minneapolis, MN, USA.

## Abstract

Transcriptome- and proteome-wide association studies (TWAS/PWAS) have proven successful in prioritizing genes and proteins whose genetically regulated expression modulates disease risk, but they ignore potential co-expression and interaction effects. To address this limitation, we introduce the co-expression-wide association study (COWAS) method, which can identify pairs of genes or proteins whose genetically regulated co-expression is associated with complex traits. COWAS first trains models to predict expression and co-expression conditional on genetic variation, and then tests for association between imputed co-expression and the trait of interest while also accounting for direct effects from each exposure. We applied our method to plasma proteomic concentrations from the UK Biobank, identifying dozens of interacting protein pairs associated with cholesterol levels, Alzheimer’s disease, and Parkinson’s disease. Notably, our results demonstrate that co-expression between proteins may affect complex traits even if neither protein is detected to influence the trait when considered on its own. We also show how COWAS can help disentangle direct and interaction effects, providing a richer picture of the molecular networks that mediate genetic effects on disease outcomes.

## Introduction

Translating genetic associations into knowledge of causal genes and proteins is a central problem in genetic epidemiology. Although genome-wide association studies (GWAS) can rapidly identify the single nucleotide polymorphisms (SNPs) and genetic loci associated with any measurable phenotype, most of the significant GWAS hits for complex traits fall outside of protein-coding regions and are thought to affect the phenome through regulatory pathways [1–6]. A popular approach for aggregating these regulatory effects into interpretable gene-level functional units is the transcriptome-wide association study (TWAS) method [7, 8]. TWAS is a two-stage framework that first trains a model to predict gene expression levels from genetic variation, thereby estimating the genetically regulated component of expression, and then tests for association between imputed expression and the trait of interest. Although most commonly applied to gene expression data, TWAS can be used with any heritable molecular phenotype. For example, proteome-wide association studies (PWAS) identify disease-relevant proteins by applying the two-stage TWAS framework to proteomic concentrations [9–11].

Many innovative methodological extensions to TWAS and PWAS have been developed since their initial introductions [12–19], with applications spanning hundreds of outcome traits [20–26]. All existing TWAS/PWAS methods, however, have a major limitation: they fail to account for correlations or interactions among the functional units being studied. In standard TWAS approaches, each gene or protein is considered independently of the rest. This marginal assumption is mathematically simple and provides for a straightforward implementation of the method, but it is biologically implausible. Moreover, discounting interaction effects in TWAS may lead to a loss of statistical power and missed biological insights when considering molecular drivers that primarily affect complex traits through synergistic pathways.

Recent methods have partially addressed the marginal limitation in TWAS by fine-mapping candidate TWAS genes to separate the effects of multiple correlated exposures [27–29]. These methods can tease out the likely causal genes within a larger set of co-expressed genes by conditioning each gene on the others. However, they do not model the genetic regulation of co-expression and cannot be used to infer the impact of gene–gene or protein–protein interactions on the outcome trait. In a separate line of research, protein–protein interaction (PPI) networks have been used to aid in the interpretation of PWAS findings [30]. Such use of PPI networks, however, still relies on the results of testing each protein individually for association with disease, and only utilizes evidence of interactions to cluster those marginal associations. Thus, no existing approaches are able to elucidate the extent to which co-expression and interactions among molecular phenotypes mediate genetic effects on complex traits.

The importance of epistasis, co-expression, and PPIs in complex disease pathogenesis has been well established and is the subject of extensive research despite the challenges of ascertaining interaction effects from genomic data [31–34]. An increasing burden of evidence also highlights the role of genetic variation in regulating gene–gene and protein–protein interactions. For example, single-cell RNA sequencing data has enabled the detection of genetic variants that significantly alter co-expression relationships [35]. More recently, a pan-cancer study demonstrated that point mutations correlate with altered, tumor-specific PPIs and can rewire interaction networks [36]. Other work used gene co-expression networks to link cancer driver genes to cancer GWAS genes, showing that common genetic variants are involved in the regulation of co-expression networks [37]. More generally, large-scale sequencing studies have established that both germline and somatic mutations are responsible for widespread perturbations in PPI networks in human diseases [38]. Such evidence suggests that it should be possible to predict the effects of genetic variation on gene or protein co-expression, and to consequently assess the association between genetically regulated co-expression and disease.

In this paper we introduce the co-expression-wide association study (COWAS) method to identify interacting genes or proteins whose genetic component of co-expression is associated with complex traits. COWAS analyzes pairs of co-expressed molecular exposures, first imputing their genetically regulated expression and co-expression, and then jointly testing for both direct effects and interaction effects on the outcome trait. We also extend COWAS to a summary statistics setting, making it easy to apply our method to any trait of interest for which GWAS summary-level data are available.

We applied COWAS to plasma proteomic concentrations from the UK Biobank (UKB) [39, 40] and large GWAS datasets for three complex traits [41–43]. We first trained imputation models for pairs of proteins with known PPIs, and then tested each well-imputed pair for association with low-density lipoprotein (LDL) cholesterol, Alzheimer’s disease (AD), and Parkinson’s disease (PD). Our results demonstrate that COWAS can successfully identify protein pairs whose co-expression impacts complex traits while at the same time disentangling their direct and interaction effects. Our approach also increases power relative to standard PWAS analyses, leading to the discovery of proteins that were missed by PWAS. Notably, we show that co-expression between proteins may affect disease risk even if neither protein influences the disease when considered on its own. Overall, our contribution provides a novel framework for studying the effects of genetically regulated co-expression on complex traits, facilitating interrogation of the phenotypic consequences of gene–gene and protein–protein interactions using GWAS summary statistics.

## Results

### Overview of COWAS

The co-expression-wide association study (COWAS) method prioritizes pairs of interacting genes or proteins whose genetically regulated expression or co-expression is significantly associated with a complex trait. Note that COWAS can be applied to either gene expression or protein expression data, but since our application concerns the proteome, we will primarily refer to protein expression throughout the rest of the paper.

**Fig. 1:**
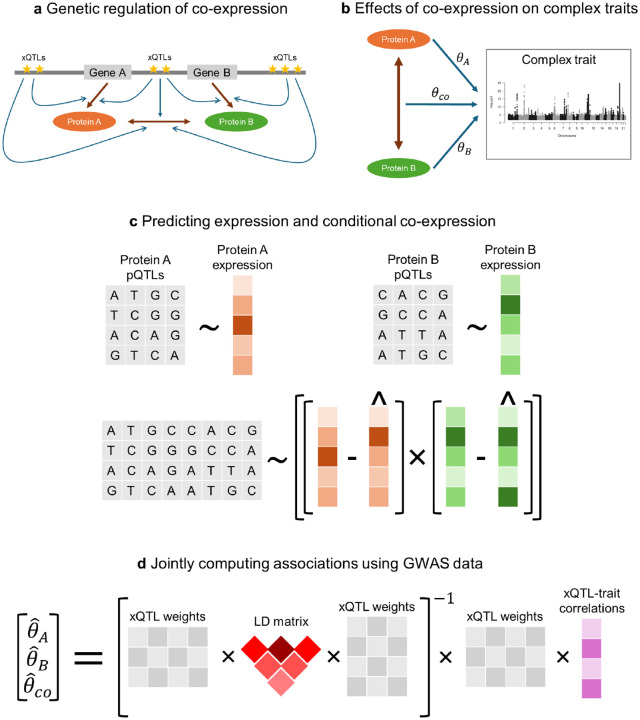
Overview of the COWAS framework. **a** Genes A and B code for proteins A and B, which interact with each other. The transcription, translation, and interaction processes are regulated by eQTLs, pQTLs, and coQTLs, respectively, which may overlap and are collectively denoted as xQTLs. **b** Proteins A and B may have direct effects on a complex trait ($\theta_{A}$ and $\theta_{B}$, respectively), but they may also impact the trait through their interactions with each other $\left(\theta_{\text{co}}\right)$. **c** The training stage of COWAS involves first building models to impute the expression levels of each protein from pQTL genotypes, and then building a third model to impute their conditional co-expression. **d** The testing stage of COWAS involves jointly estimating direct and interaction effects on a complex trait of interest using the fitted model weights from the training stage, an LD reference panel, and GWAS summary statistics for the outcome trait.

The key motivation behind our approach is the observation that genetic variation modulates not only protein expression, but also protein co-expression (Figure 1a). We refer to genetic variants associated with co-expression as co-expression quantitative trait loci (coQTLs) [35], analogously to how variants associated with gene expression are termed expression quantitative trait loci (eQTLs) and variants associated with protein expression are termed protein quantitative trait loci (pQTLs). A variant can belong to one or more of these xQTL classes, but we assume that a coQTL is most likely also an eQTL or a pQTL. Furthermore, we consider co-expression to be a proxy for interaction effects. Although gene–gene and protein–protein interactions are not directly measured in large biobank studies such as the UKB, co-variation of protein abundance is an accurate proxy for PPIs because interacting protein pairs are known to be highly co-expressed [36, 44]. COWAS leverages pQTL data to learn the patterns of genetic regulation underlying protein expression and co-expression, and ultimately estimates the direct and interaction effects of genetically regulated expression on a complex trait of interest (Figure 1b).

The COWAS framework is comprised of a training stage (Figure 1c) and a testing stage (Figure 1d). The training stage must be performed on individual-level genotype and expression data. First, models are trained to predict the expression levels of each protein from its pQTLs. Next, the measured and imputed expression levels are used to estimate a quantity derived from the conditional Pearson correlation coefficient. Finally, a third model is trained to impute this quantity from the union of all considered pQTLs. Predictions from the co-expression model have the desired property of representing the correlation between the two proteins’ expression levels conditioned on genetic information. Our method exploits the properties of conditional covariance to remove the components of co-expression that are explained by genetic effects on mean expression levels or by factors unrelated to genetics, allowing us to focus on how genetic variation modulates the amount of correlation between the two exposures. Explicitly modeling the conditional correlation of expression is the primary innovation of COWAS, because it enables our approach to incorporate the genetic component of gene or protein co-expression into an association testing framework.

The testing stage of COWAS is typically performed using fitted model weights from the training stage, a linkage disequilibrium (LD) reference panel, and summary-level GWAS data for the outcome trait of interest (Figure 1d). Here three effect sizes are jointly estimated: the direct effect of the first protein’s genetically regulated expression on the trait $\left(\theta_{A}\right)$, the direct effect of the second protein’s genetically regulated expression on the trait $\left(\theta_{B}\right)$, and the effect of their genetically regulated co-expression on the trait $\left(\theta_{co}\right)$. Note that $\theta_{A}$ and $\theta_{B}$ are distinct from the marginal effects obtained through standard TWAS or PWAS, since here the three effect sizes are estimated together in a joint model. As a result, each effect size is conditional on the other two.

Several hypothesis tests can be performed with these estimated effect sizes and their standard errors. The COWAS global test determines if the protein pair has an overall effect on the outcome trait, potentially boosting power relative to marginal TWAS/PWAS analyses of each exposure. Alternatively, we can test the effect size estimates individually in order to disentangle the impact of each protein’s genetically regulated expression from the impact of their genetically regulated co-expression. In particular, the COWAS interaction test determines if co-expression has an effect on the outcome trait while accounting for direct effects from both exposures. This flexibility and increased statistical power enable COWAS to identify novel disease-relevant genes or proteins and aid in the interpretation of GWAS findings. Significant COWAS protein pairs can then be visualized as an interaction network in order to highlight protein complexes that mediate genetic risk on the outcome trait.

### Accurately imputing genetically regulated co-expression

We trained COWAS models to predict protein expression and co-expression using genotypes and proteomic concentrations from the UKB Pharma Proteomics Project [40]. After quality control, we retained 2,833 proteins coded by autosomal genes. Since training imputation models for each of the $\left(\begin{array}{c} 2,833\\ 2 \end{array}\right)=4,\;011,\;528$ possible protein pairs would have been computationally infeasible, we restricted our analysis to pairs with some prior evidence of PPIs, as listed in the Human Integrated Protein–Protein Interaction rEference (HIPPIE) database [45]. In total, we trained COWAS models using UKB genotypes and normalized protein abundance residuals for 26,433 protein pairs.

**Fig. 2:**
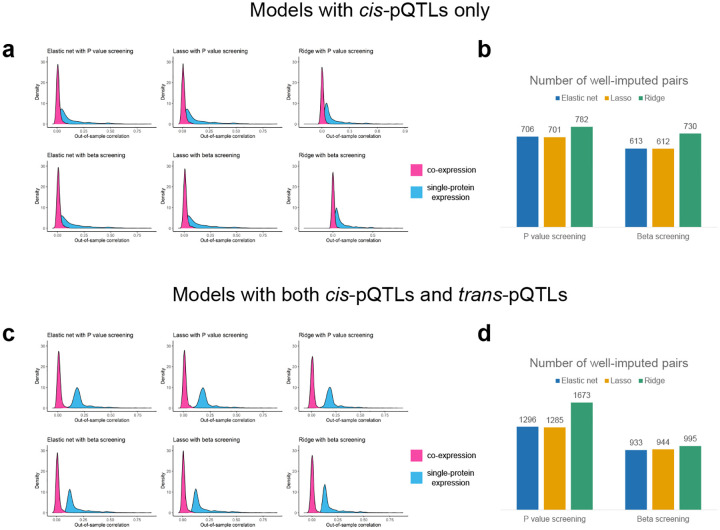
Performance metrics for COWAS models trained on UKB data. **a,c** Density plots of the out-of-sample correlation between estimated and imputed co-expression (in pink), and between measured and imputed single-protein expression (in blue). **b,d** Counts of the numbers of protein pairs in which all three prediction models had an out-of-sample correlation greater than 0.03. Models in **a** and **b** were trained with only *cis*-pQTLs as predictors. Models in **c** and **d** were trained with both *cis*-pQTLs and *trans*-pQTLs as predictors.

To ensure that COWAS can accurately predict genetically regulated co-expression, we explored the out-of-sample imputation performance of several regression methods (Figure 2). We considered penalized linear regression models with either an elastic net penalty, a lasso penalty, or a ridge penalty. For each of these three model types, we pre-screened genetic variants using either the P values or the effect sizes of their association with each protein’s expression. Additionally, we also considered the extent to which including both local pQTLs (*cis*-pQTLs) and distant pQTLs (*trans*-pQTLs) improved model imputation performance relative to only including *cis*-pQTLs.

Our results show that accurate imputation is more challenging for protein co-expression than for the expression of individual proteins. Across all of the model types we considered, the median out-of-sample correlation between estimated and imputed co-expression was always lower than between measured and imputed single-protein expression (Figure 2a, Figure 2c, and Supplementary Data 1). This was expected, since interaction effects are known to be more difficult to detect than main effects, with considerably larger sample sizes being needed for the same level of power or prediction quality. Interestingly, including *trans*-pQTLs in addition to *cis*-pQTLs significantly increased the imputation quality for single-protein models, but it did not have a pronounced effect on the performance of co-expression models (Figure 2a and Figure 2c). This suggests that *trans*-pQTLs only weakly regulate PPIs, with the bulk of heritability in co-expression stemming from local genetic variation. However, it is also possible that *trans*-coQTLs may not overlap with *trans*-pQTLs. Since we pre-screened genetic variants based on the strength of their association with the individual proteins in each pair, the inclusion of distant variants primarily increases the number of strong pQTLs present in each model and may not necessarily increase the number of strong coQTLs.

Next, we filtered the protein pairs to those in which all three imputation models yielded an out-of-sample correlation greater than 0.03 (Figure 2b and Figure 2d). Among these well-imputed pairs, lasso regression with *cis*-pQTLs pre-screened by their effect sizes achieved the highest mean out-of-sample $R^{2}$ for predicting co-expression (mean $R^{2}=0.0038$, Supplementary Data 1). On the other hand, ridge regression with both *cis*-pQTLs and *trans*-pQTLs pre-screened by their P values yielded the greatest number of well-imputed protein pairs (Figure 2d and Supplementary Data 1). We decided to use the former approach in our main analyses in order to maximize the imputation quality of conditional co-expression. Model performance metrics for every combination of protein pair and model type are provided in Supplementary Data 2–13.

### COWAS identifies co-expressed proteins associated with complex traits

Having shown that COWAS is able to accurately impute both single-protein expression and protein co-expression, we applied it to three complex trait outcomes: low-density lipoprotein (LDL) cholesterol, Alzheimer’s disease (AD), and Parkinson’s disease (PD). For each trait, we downloaded summary-level data from the largest publicly available GWAS study [41–43]. To ensure complete overlap between the genetic variants included in the imputation models and the GWAS data, we re-trained COWAS models for each trait using only the intersection of variants found in both the UKB genotype data and the trait’s GWAS. We also re-assessed the out-of-sample predictive performance of each model separately for each trait and only kept pairs with sufficiently high imputation accuracy, thus guaranteeing that differences between the GWAS datasets do not negatively impact the validity of association testing. As a result, the numbers of considered protein pairs somewhat differed among the three traits. For LDL cholesterol 613 pairs were accurately imputed, for AD there were only 564 well-imputed pairs, and for PD we retained 592 well-imputed pairs (Supplementary Data 14–16).

To compare our new approach with currently available methods, we also performed a standard PWAS analysis for each protein included in the COWAS analyses. The same training samples, model types, and variant screening strategies were applied for both COWAS and PWAS. Namely, we selected the top 100 pQTLs for each protein by their association effect sizes and used them as features in linear regression models with a lasso penalty. Full imputation performance metrics for all analyzed proteins and outcome traits are provided in Supplementary Data 14–16. We also used the same LD reference panel derived from UKB data when computing effect sizes in both COWAS and PWAS. To account for multiple testing in COWAS, we performed a Bonferroni correction on the number of well-imputed protein pairs. Similarly, in standard PWAS we performed a Bonferroni correction on the number of well-imputed proteins.

Our results demonstrate that COWAS is able to detect PPIs with a significant genetically regulated effect on complex traits. For LDL cholesterol, which had the best-powered GWAS of the three traits we considered, we identified 38 protein pairs with a significant co-expression effect (Supplementary Figure 1). Of these protein pairs, 24 had at least one protein that was also identified by a standard PWAS analysis, while the rest were uniquely identified by our method. We also performed a global test on each pair to assess whether it has an overall effect on LDL cholesterol, which yielded 116 significant pairs. As expected, nearly all of those pairs contained at least one protein that was also detected by PWAS. However, the COWAS global test did identify 12 pairs with a significant effect on LDL cholesterol in which neither protein was significant when considered on its own, and 5 of those pairs did not even have a significant interaction term. This suggests that explicitly modeling co-expression can boost power relative to standard marginal tests, even when there is no statistically significant effect of co-expression on the outcome trait.

COWAS can also help disentangle the effects of interacting groups of proteins on complex traits, thereby providing a richer picture of the functional consequences of genetically regulated molecular phenotypes. By visualizing significant COWAS pairs as a network, we identified complexes of mutually interacting proteins that modulate LDL cholesterol levels (Supplementary Figure 2). Moreover, our method can reveal when the effect of genetically regulated co-expression on a complex trait is in the opposite direction relative to the effects of the interacting proteins themselves. For example, we found that APOE and PLTP both decrease LDL cholesterol levels, while their co-expression was associated with increased LDL cholesterol levels (Supplementary Figure 3). In other cases, the direct and interaction effects may all be in the same direction, such as observed for the effects of APOE and AGRN on LDL cholesterol. Full results for LDL cholesterol, including the estimated effect sizes and standard errors within each pair, are provided in Supplementary Data 14.

### COWAS boosts power and corroborates known PPIs driving Alzheimer’s disease risk

**Fig. 3:**
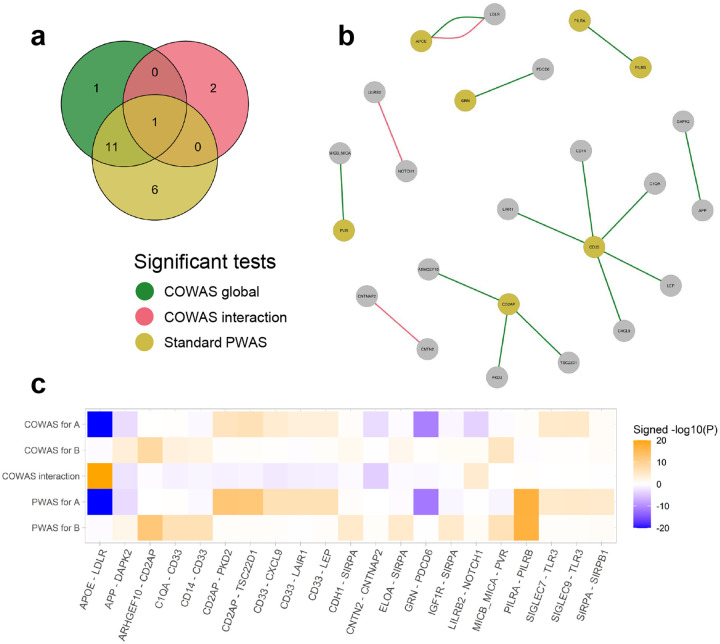
COWAS and PWAS results for Alzheimer’s disease. **a** A Venn diagram displaying the numbers of protein pairs identified as significant for AD by the COWAS global test (green), the COWAS interaction test (pink), or a standard PWAS analysis (yellow). Here “standard PWAS” refers to pairs in which at least one of the proteins was identified by PWAS. **b** A network diagram showing all of the protein pairs identified as significant for AD by either the COWAS global test (green edges) or the COWAS interaction test (pink edges). Node colors indicate whether each protein was identified as significant for AD by standard PWAS (yellow) or not (gray). **c** A heat map displaying signed $-{\text{log}}_{10}(P)$ values from COWAS single-protein and interaction tests as well as from a standard PWAS analysis for all pairs included in the Venn diagram. A and B refer to the first and second proteins listed in each pair, respectively. To facilitate visualization, the $-{\text{log}}_{10}(P)$ values were capped at 20.

We identified fewer significant protein pairs for AD compared to LDL cholesterol, but this was expected due to the lower power of the corresponding GWAS study. Yet here again, both the COWAS global test and the COWAS interaction test were able to detect significant protein pairs missed by standard PWAS (Figure 3a). Our method can also provide additional insights even in pairs where one protein was identified by PWAS. For example, we discovered 5 proteins that jointly modulate AD risk together with CD33 (Figure 3b). Although CD33 itself was identified by standard PWAS, our application of COWAS reveals a fuller picture of the molecular pathways underlying AD.

Notably, the COWAS global test identified the pair comprised of amyloid-beta precursor protein (APP) and death-associated protein kinase 2 (DAPK2) as significant for AD (P=1.25e-05), while a standard PWAS analysis failed to identify either of these proteins (P=7.72e-04 for APP and P=1.31e-02 for DAPK2). APP is concentrated in the synapses of neurons and is the precursor molecule for the generation of amyloid beta (Aβ), which contributes to the formation of amyloid plaques—a hallmark pathology in AD [46–48]. Yet despite the central role of APP in Alzheimer’s pathogenesis, standard PWAS lacked the power to identify it in our dataset. On the other hand, COWAS was able to boost power and attain statistical significance by jointly considering APP and a member of the DAPK family, which has also been previously implicated in late-onset AD [49].

Furthermore, COWAS discovered a highly significant effect of the interaction between APOE and LDLR on AD risk (P<1e-50). Although APOE was also highly significant according to a standard PWAS analysis (P<1e-50), LDLR was not (P=0.48). This result is notable, because LDLR is known to be a receptor for APOE that preferentially binds lipidated APOE particles and plays an important role in Aβ clearance [50, 51]. Our results are consistent with this mechanistic explanation, since we found APOE and its interaction with LDLR to have opposite effects on AD (Figure 3c). Thus, COWAS provides strong support to the hypothesis that APOE and LDLR have a synergistic effect in Alzheimer’s pathogenesis, even after accounting for the direct effect of APOE on AD risk.

The other two significant interactions implicated by COWAS for AD are also likely true positives, further confirming the sensitivity and power of our approach. We identified a significant effect of the interaction between LILRB2 and NOTCH1 on AD (P=5.73e-05), whereas standard PWAS failed to identify either protein (P=0.80 for LILRB2 and P=0.13 for NOTCH1). LILRB2 is a neuronal cell surface receptor that interacts with Aβ and is being studied as a promising therapeutic target for AD [52, 53], while NOTCH1 has been found to be differentially expressed in Alzheimer’s patients [54] and is potentially involved in neurodegeneration-related cell signaling disruptions [55]. Finally, the COWAS interaction test also discovered a significant effect of co-expression between CNTN2 and CNTNAP2 on AD (P=8.42e-05), whereas standard PWAS again failed to detect either protein as significant (P=0.94 and P=0.38, respectively). The mechanisms by which these proteins are involved in Alzheimer’s pathology have not yet been thoroughly studied, but earlier genetic and functional genomic evidence indicates that they might play a role [56]. Full results for AD, including the estimated effect sizes and standard errors within each pair, are provided in Supplementary Data 15.

### Co-expression analysis identifies SNCA interactions in Parkinson’s disease pathogenesis

**Fig. 4:**
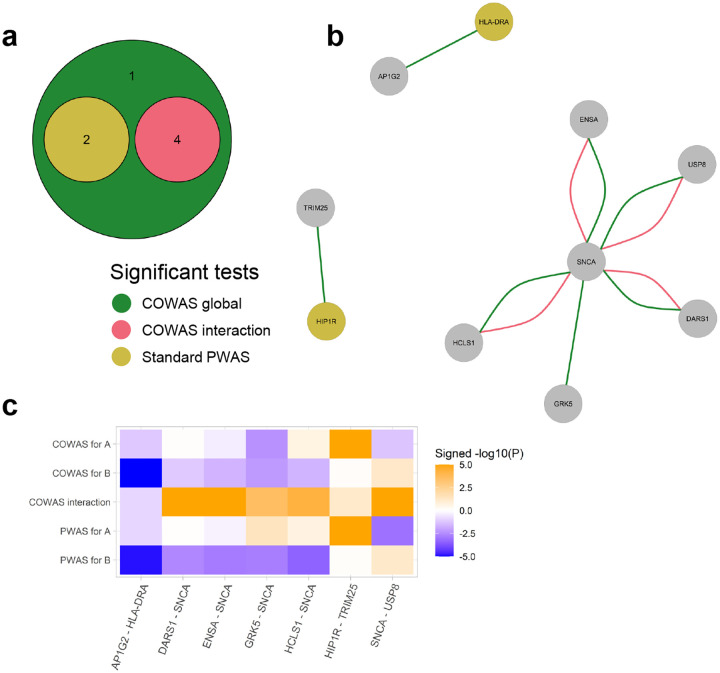
COWAS and PWAS results for Parkinson’s disease. **a** A Venn diagram displaying the numbers of protein pairs identified as significant for PD by the COWAS global test (green), the COWAS interaction test (pink), or a standard PWAS analysis (yellow). Here “standard PWAS” refers to pairs in which at least one of the proteins was identified by PWAS. **b** A network diagram showing all of the protein pairs identified as significant for PD by either the COWAS global test (green edges) or the COWAS interaction test (pink edges). Node colors indicate whether each protein was identified as significant for PD by standard PWAS (yellow) or not (gray). **c** A heat map displaying signed $-{\text{log}}_{10}(P)$ values from COWAS single-protein and interaction tests as well as from a standard PWAS analysis for all pairs included in the Venn diagram. A and B refer to the first and second proteins listed in each pair, respectively. To facilitate visualization, the $-{\text{log}}_{10}(P)$ values were capped at 5.

For PD the COWAS global test identified all of the protein pairs that were discovered by the COWAS interaction test or by a standard PWAS analysis (Figure 4a). In addition to those pairs, the COWAS global test also uniquely identified an effect of GRK5 and SNCA on PD (P=4.81e-06). This pair was not significant according to the COWAS interaction test (P=2.75e-04) or according to standard PWAS (P=0.04 for GRK5 and P=1.65e-03 for SNCA). However, note that both of these proteins have been previously implicated in PD pathogenesis. Alpha-synuclein (SNCA) regulates the release of neurotransmitters from the axon terminals of presynaptic neurons, and insoluble forms of SNCA accumulate in the form of Lewy bodies, leading to nerve cell death and the development of PD symptoms [57–59]. As for GRK5, some evidence suggests that it plays a role in the pathogenesis of sporadic forms of PD [60]. These results further highlight the ability of COWAS to boost power relative to marginal approaches such as PWAS.

Interestingly, all four of the significant co-expression effects on Parkinson’s risk that were identified by COWAS are comprised of SNCA interacting with some other protein (Figure 4b). In particular, the COWAS interaction test identified significant effects on PD from genetically regulated co-expression between SNCA and DARS1 (P=1.21e-13), SNCA and ENSA (P=1.03e-08), SNCA and HCLS1 (P=5.50e-05), and SNCA and USP8 (P=3.64e-07). Note that co-expression between SNCA and each of these four proteins has a positive effect on PD even though the effect of SNCA itself is negative (Figure 4c). This suggests that a genetically regulated escalation of co-expression between SNCA and each of these proteins elevates PD risk, illustrating potential avenues for therapeutic intervention.

None of the four proteins whose interaction with SNCA had an effect on PD were significant according to a standard PWAS analysis, with marginal PWAS P values ranging from P=0.88 to P=0.07 (Supplementary Data 16). However, the COWAS discoveries are reasonable in light of previous research. For example, USP8 is a deubiquitinase that has also been found in Lewy bodies and plays a role in determining SNCA levels [61, 62]. ENSA has been shown to interfere with SNCA self-assembly and thereby alleviate its neurotoxicity [63], and variants in HCLS1 binding protein 3 were found to be associated with the related condition of essential tremor (but not PD itself) [64]. We are not aware of any existing evidence for the role of DARS1 in Parkinson’s pathogenesis, but its identification by COWAS points to a potential avenue for further research. Full results for PD, including the estimated effect sizes and standard errors within each pair, are provided in Supplementary Data 16.

## Discussion

In this paper we introduced the co-expression-wide association study (COWAS) method, the first statistical framework for identifying gene or protein pairs whose genetically regulated interactions are associated with complex traits. COWAS extends the two-stage least squares approach underlying TWAS/PWAS by explicitly imputing the conditional correlation between pairs of exposures, which we interpret as a proxy for genetically regulated gene–gene or protein–protein interactions. This enables COWAS to jointly test for direct and interaction effects of genetically regulated expression on a complex trait of interest, thereby boosting power relative to existing methods and helping to disentangle the functional mechanisms by which molecular exposures influence the outcome trait. We also extended COWAS to a summary statistics setting, making it easy to apply our method to any trait for which GWAS summary data are available.

In our application of COWAS to the UKB Pharma Proteomics Project dataset, we first explored the performance of different regression models for imputing genetically regulated co-expression and then applied our method to identify protein pairs associated with three complex traits. Our method was able to discover biologically relevant co-expressed proteins for all three traits, highlighting the importance of interaction effects in driving complex disease risk. Notably, COWAS identified a number of protein pairs with a significant interaction term in which neither protein had a significant effect when analyzed independently via standard PWAS. These results underscore the importance of considering interaction effects in future research, since the marginal TWAS/PWAS approaches currently used to analyze molecular phenotypes may be missing important sources of signal. Moreover, our results demonstrate how COWAS can be used to implicate groups of proteins in complex disease risk and distinguish between direct and interaction effects, providing a more complete picture of the molecular pathways that mediate genetic risk on downstream traits.

Notwithstanding the many advantages of COWAS, our approach has several limitations. First of all, COWAS only considers one pair of molecular units at a time. Although these pairwise results can be visualized as a network, COWAS does not simultaneously model the genetic regulation of entire protein complexes. Proteins may interact in larger, multi-protein interaction networks with nontrivial topological structures [65–67], and it is possible that genetic variation may impact such higher-order network properties. Extending COWAS to allow for other types of interactions among more than two exposures at a time could illuminate additional disease-relevant genes and proteins, but it is not obvious how to do so in a computationally efficient way. Furthermore, we found that the predictive capacity of protein co-expression imputation models is lower than that of expression imputation models for individual proteins. This was expected given the difficulty of ascertaining interaction effects in general, yet even so we were able to obtain sufficiently good imputation quality for over a thousand protein pairs. However, more work could be done to explore different machine learning algorithms for training co-expression imputation models. Finally, we only considered individuals of a single genetic ancestry in this study. Since transcriptome and proteome imputation models are generally not portable across ancestry groups [68–71], we subset the UKB data to the largest genetically-inferred ancestry subgroup, which roughly corresponds to White British individuals, and correspondingly used GWAS studies conducted on European individuals for our three outcome traits. An extension of COWAS to handle multiple genetic ancestries and admixed individuals would expand the diversity and relevance of its applications.

The field of human genetics has historically focused on studying linear, marginal effects. This is exemplified by the popularity of GWAS and TWAS/PWAS analyses, which only consider one genetic variant or one functional molecular unit at a time. By providing a simple yet powerful approach for analyzing genetically regulated gene or protein co-expression using existing biobank data, our work joins the growing body of evidence emphasizing the limitations of this historical paradigm. The COWAS method exhibits high statistical power, provides flexibility in modeling direct and interaction effects, and is easy to use. We envision that COWAS, along with its future improvements and extensions, will enhance the interpretation of genomic findings and lead to the discovery of new biological insights and therapeutic targets.

## Methods

### Modeling genetically regulated co-expression

The COWAS method is applied to one outcome trait and two molecular exposures at a time. Let $A,B\in R^{n}$ denote the expression or abundance levels of the two exposures, as measured in n individuals. Further, let $Z_{A}\in R^{n\times p_{A}}$ be the genotype matrix of $p_{A}$ xQTLs for exposure A, which were genotyped in the same set of individuals. Similarly, let $Z_{B}\in R^{n\times p_{B}}$ be the genotype matrix of $p_{B}$ xQTLs for exposure B, and let $Z\in R^{n\times p}$ be the joint matrix of all p xQTLs, where p is the number of unique variants in the union of xQTLs for the two exposures. (If there is no overlap among the xQTLs for the two exposures, then $p=p_{A}+p_{B}$.) Finally, let $Y\in R^{n}$ be the outcome trait of interest. All of these vectors and each column of these matrices are assumed to be centered around 0 and scaled to have a variance of 1.

Just like in standard TWAS or PWAS, we assume that the mean genetically regulated expression of each molecular exposure can be modeled as a linear combination of its xQTL genotypes. That is,

(1) $$A=\gamma_{A}+Z_{A}\beta_{A}+\varepsilon_{A},$$

(2) $$B=\gamma_{B}+Z_{B}\beta_{B}+\varepsilon_{B}.$$

Here $\beta_{A}\in R^{p_{A}}$ and $\beta_{B}\in R^{p_{B}}$ are unknown xQTL weights, while $\gamma_{A}\in R$ and $\gamma_{B}\in R$ are unknown intercepts. The error terms $\varepsilon_{A}$ and $\varepsilon_{B}$ are assumed to be normally distributed.

What sets COWAS apart from previous methods, however, is that we also model the genetically regulated co-expression of the two functional units instead of analyzing them independently of each other. The most popular metric for co-expression is the Pearson correlation between measured expression levels [72, 73]. Therefore, genetically regulated co-expression should be defined as the Pearson correlation conditional on genetic information. Formally, we define the genetically regulated co-expression of A and B as

(3) $$\text{Corr}\left(A,B|Z_{A},Z_{B}\right)=\frac{\text{Cov}\left(A,B|Z_{A},Z_{B}\right)}{\sqrt{\text{Var}\left(A|Z_{A}\right)\text{Var}\left(B|Z_{B}\right)}},$$

where the conditional covariance between A and B is

(4) $$\text{Cov}\left(A,B|Z_{A},Z_{B}\right)=E\left(\left(A-E\left(A|Z_{A}\right)\right)\left(B-E\left(B|Z_{B}\right)\right)|Z_{A},Z_{B}\right).$$

To simplify estimation of this quantity, we make the assumption that $\text{Var}\left(A|Z_{A}\right)$ and $\text{Var}\left(B|Z_{B}\right)$ are both constant. In other words, we assume that genetic variants only regulate the mean of each molecular phenotype and their covariance, but not their individual variances. Although not exactly true from a biological perspective, this simplifying assumption is reasonable because any effects of genetic variation on the variance of protein concentrations are likely to be much smaller than the effects of genetic variation on the quantities we are considering.

Using these definitions and assumptions, we can approximate the conditional correlation of A and B as

(5) $$\text{Corr}\left(A,B|Z_{A},Z_{B}\right)\;\propto \text{Cov}\left(A,B|Z_{A},Z_{B}\right)$$

(6) $$=E\left(\left(A-E\left(A|Z_{A}\right)\right)\left(B-E\left(B|Z_{B}\right)\right)|Z_{A},Z_{B}\right)$$

(7) $$\approx E\left((A-\hat{A})(B-\hat{B})|Z_{A},Z_{B}\right)$$

where $\hat{A}=Z_{A}{\hat{\beta }}_{A}$ and $\hat{B}=Z_{B}{\hat{\beta }}_{B}$ are the genetically imputed expression levels of the two exposures. Thus, the appropriate way to model genetically regulated co-expression is

(8) $$\left(A-\hat{A}\right)\left(B-\hat{B}\right)=\gamma_{\text{co}}+Z\beta_{\text{co}}+\varepsilon_{\text{co}},$$

where $\beta_{\text{co}}\in R^{p}$ is the unknown vector of coQTL weights and $\gamma_{\text{co}}\in R$ is an unknown intercept. The random error term $\varepsilon_{\text{co}}$ is again assumed to be normally distributed. For conciseness, let $C=(A-\hat{A})(B-\hat{B})$ and $\hat{C}=Z{\hat{\beta }}_{\text{co}}$.

We can interpret ${\hat{\beta }}_{\text{co}}$ as representing the effects of genetic information on the correlation between A and B. In general, correlation between A and B may be due to some combination of the following three factors:

1. Genetic effects on the mean expression levels of the two proteins. In other words, co-expression can be induced through correlation between the genetically regulated expression levels $E\left(A|Z_{A}\right)$ and $E\left(B|Z_{B}\right)$.
2. Genetic effects that modulate the correlation between A and B. That is, genetic variation can influence the level of correlation between $\varepsilon_{A}$ and $\varepsilon_{B}$.
3. Factors unrelated to genetics. For example, a shared tissue environment or various other environmental effects may cause A and B to be correlated.

Our formulation of conditional covariance in Equation (7) effectively removes the first factor, so what remains may be some combination of the second and third factors. However, the effects of any factors unrelated to genetics should be constant with respect to genetic variation, and so they will be captured by the intercept term $\gamma_{\text{co}}$. Notice that we estimate this intercept term and then discard it before imputing $\hat{C}$, thereby removing environmental effects on co-expression. In the end, this procedure for estimating $\hat{C}$ isolates the genetic component of co-expression.

Next, we estimate the effect of genetically regulated co-expression on the outcome trait (Y) while accounting for direct effects of A and B on Y. We assume that the outcome trait depends on a linear combination of the genetically regulated expression levels of both molecular exposures and their genetically regulated co-expression. Formally, our model for the outcome trait is

(9) $$Y=\hat{A}\theta_{A}+\hat{B}\theta_{B}+\hat{C}\theta_{\text{co}}+\varepsilon_{Y},$$

where $\theta_{A},\theta_{B},\theta_{\text{co}}\in R$ are unknown scalars and $\varepsilon_{Y}$ is a normally distributed, independent error term. The ultimate goal of COWAS is to estimate $\theta_{A},\theta_{B}$, and $\theta_{\text{co}}$ and to test each of them for statistical significance. We will also derive a global test to determine if the model in Equation (9) is significantly better than a null model.

### Two-sample model estimation and hypothesis testing

In practice, the COWAS models are estimated in a two-sample setting akin to standard TWAS and PWAS. Suppose we have two nonoverlapping, individual-level datasets from the same population with sample sizes $n_{1}$ and $n_{2}$. Genotypes for all p xQTLs are available in both datasets, but the molecular exposures A,B are only measured on the $n_{1}$ samples in the first dataset, while the outcome trait Y is only measured on the $n_{2}$ samples in the second dataset.

In the model training stage, COWAS first trains models to estimate $\beta_{A}$ and $\beta_{B}$ using data from the first dataset. Then it imputes expression for each exposure on that same dataset. That is, we compute

(10) $$\hat{A}=Z_{A}{\hat{\beta }}_{A},$$

(11) $$\hat{B}=Z_{B}{\hat{\beta }}_{B},$$

using the same $n_{1}$ individuals used for model training. Next, COWAS computes the quantity $C=(A-\hat{A})(B-\hat{B})$. This quantity is then used as the outcome for training the model in Equation (8), yielding the fitted weights ${\hat{\beta }}_{\text{co}}$.

In the testing stage, the fitted weights ${\hat{\beta }}_{A},$ ${\hat{\beta }}_{B}$, and ${\hat{\beta }}_{\text{co}}$ are used to impute expression and co-expression for the $n_{2}$ samples in the second dataset. That is, we compute

(12) $${\hat{A}}^{*}=Z_{A}^{*}{\hat{\beta }}_{A},$$

(13) $${\hat{B}}^{*}=Z_{B}^{*}{\hat{\beta }}_{B},$$

(14) $${\hat{C}}^{*}=Z^{*}{\hat{\beta }}_{\text{co}},$$

where the * symbol is used to distinguish quantities measured or imputed in the outcome dataset from those in the expression dataset. Finally, we fit the outcome trait model

(15) $$Y={\hat{A}}^{*}\theta_{A}+{\hat{B}}^{*}\theta_{B}+{\hat{C}}^{*}\theta_{\text{co}}+\varepsilon_{Y}$$

to estimate each of its coefficients and their standard errors. Any linear model hypothesis tests can be performed on the estimated coefficients ${\hat{\theta }}_{A},$ ${\hat{\theta }}_{B}$, and ${\hat{\theta }}_{\text{co}}$. In this study, we primarily consider an interaction test and a global test.

#### Interaction test:

To determine if co-expression has an effect on the outcome trait, we test the hypothesis $H_{0}:\theta_{\text{co}}=0$ against its two-sided alternative using a Wald test. Namely, the test statistic is $w={\left({\hat{\theta }}_{\text{co}}\right)}^{2}/\text{Var}\left({\hat{\theta }}_{\text{co}}\right)$, which asymptotically follows a $\chi^{2}$ distribution with 1 degree of freedom under $H_{0}$.

#### Global test:

To determine if the two exposures have an overall effect on the outcome trait, we test whether the model in Equation (15) fits the data better than an intercept-only model using an F test. Namely, the test statistic is $f=\frac{\text{RS}S_{\text{null}}-\text{RS}S}{\left(n_{2}-1\right)-\left(n_{2}-4\right)}/\frac{\text{RS}S}{n_{2}-4}=\frac{n_{2}-4}{3\text{RS}S}\left(n_{2}-1-\text{RS}S\right)$, where RSS is the residual sum of squares from the COWAS model in Equation (15) and $RSS_{\text{null}}=n_{2}-1$ is the residual sum of squares from an intercept-only model. f follows an F distribution with $\left(3,n_{2}-4\right)$ degrees of freedom under the null hypothesis.

The interaction test and the global test can help to disentangle the effects of co-expression from the direct effects of the individual exposures. If the global test rejects its null hypothesis but the interaction test does not, we can conclude that the molecular exposures directly influence the outcome trait. Our implementation of COWAS in R also provides P values for Wald tests on $\theta_{A}$ and $\theta_{B}$, enabling users to test whether each exposure has a significant effect while accounting for the other exposure and the co-expression term.

### Extension of COWAS for use with GWAS summary data

In this section we extend the association testing stage of COWAS so that it can be performed with summary-level GWAS data. The formulas we derive here only require fitted weights for expression and co-expression imputation models, Z scores from a GWAS for the outcome trait, and an LD reference panel. Note that we used this summary-level version of COWAS to obtain all of the results reported in this paper.

Let ${\hat{\beta }}_{A},{\hat{\beta }}_{B},{\hat{\beta }}_{\text{co}}\in R^{p}$ be the trained model weights for molecular phenotypes A,B and their co-expression, respectively. Note that, unlike in the individual-level formulation, the dimensions of all three weight vectors must match; this can be ensured by padding ${\hat{\beta }}_{A}$ and ${\hat{\beta }}_{B}$ with zeros. We will denote the joint matrix of all model weights by $\hat{\beta }=\left({\hat{\beta }}_{A},{\hat{\beta }}_{B},{\hat{\beta }}_{\text{co}}\right)\in R^{p\times 3}$.

Furthermore, let $z_{1},\ldots ,z_{p}\in R$ be Z scores from a GWAS study for the outcome trait of interest (Y) for the same set of p genetic variants. We assume that the GWAS was conducted in a population of the same genetic ancestry as the population used to train COWAS model weights. Importantly, reference and effect alleles must be consistent between the GWAS summary data and the COWAS weights. Our implementation of COWAS automatically checks for allele consistency, flips GWAS Z scores when necessary, and removes variants that cannot be harmonized. Next, COWAS converts the GWAS Z scores to pseudocorrelation estimates. This is done by relying on the monotonic relationship between Z scores and correlations [74], leading to the following formula for the pseudocorrelation between Y and variant i:

(16) $${\hat{c}}_{i}=\frac{z_{i}}{\sqrt{n^{\prime }-1+z_{i}^{2}}},$$

where $n^{\prime }$ is the sample size of the GWAS cohort for the outcome trait.

Finally, let $G\in R^{m\times p}$ be a genotype matrix for m individuals and the same set of p variants included in the COWAS models. We assume that these m individuals are of the same genetic ancestry as those used to train the COWAS model weights and conduct the outcome trait GWAS. Moreover, we assume that each column of G has been centered around 0 and scaled to a variance of 1. An LD reference panel represents correlations among genetic variants, so we compute it from G as

(17) $$\hat{D}=\frac{1}{m}G^{⊤}G.$$

Now we will derive an estimator for $\theta ={\left(\theta_{A},\theta_{B},\theta_{\text{co}}\right)}^{⊤}$ in terms of the trained COWAS model weights $\hat{\beta }$, the variant-outcome pseudocorrelation vector $\hat{c}={\left({\hat{c}}_{1},\ldots ,{\hat{c}}_{p}\right)}^{⊤}$, and the LD reference panel $\hat{D}$. Suppose that ${\hat{X}}^{*}=\left({\hat{A}}^{*},{\hat{B}}^{*},{\hat{C}}^{*}\right)\in R^{n_{2}\times 3}$ is the matrix of imputed expression and co-expression in an individual-level dataset for the outcome trait, so that Equation (15) can be rewritten as $Y={\hat{X}}^{*}\theta +\varepsilon_{Y}$. This is a multiple linear regression model, so the ordinary least squares estimator of θ is

(18) $$\hat{\theta }\;={\left({\left({\hat{X}}^{*}\right)}^{⊤}{\hat{X}}^{*}\right)}^{-1}{\left({\hat{X}}^{*}\right)}^{⊤}Y$$

(19) $$={\left({\left(Z^{*}\hat{\beta }\right)}^{⊤}Z^{*}\hat{\beta }\right)}^{-1}{\left(Z^{*}\hat{\beta }\right)}^{⊤}Y$$

(20) $$={\left({\hat{\beta }}^{⊤}\frac{Z^{*⊤}Z^{*}}{n_{2}}\hat{\beta }\right)}^{-1}{\hat{\beta }}^{⊤}\frac{Z^{*⊤}Y}{n_{2}}.$$

Observe that $\frac{Z^{*⊤}Z^{*}}{n_{2}}$ is a matrix of correlations among the xQTLs in $Z^{*}$, so we can estimate it with $\hat{D}$. Moreover, $\frac{Z^{*T}Y}{n_{2}}$ is a vector of correlations between each variant and Y, so we can estimate it with $\hat{c}$. Therefore, the effects of expression and co-expression on the outcome trait are jointly estimated by

(21) $$\hat{\theta }={\left({\hat{\beta }}^{⊤}\hat{D}\hat{\beta }\right)}^{-1}{\hat{\beta }}^{⊤}\hat{c}.$$

Similarly, the variance of $\hat{\theta }$ can be estimated in terms of $\hat{\theta },\hat{\beta },\hat{D}$, and $\hat{c}$. The residual sum of squares for $Y={\hat{X}}^{*}\theta +\varepsilon_{Y}$ is

(22) $$\text{RSS}\;={\left\|Y-{\hat{X}}^{*}\hat{\theta }\right\|}^{2}$$

(23) $$=Y^{⊤}Y-2Y^{⊤}{\hat{X}}^{*}\hat{\theta }+{\hat{\theta }}^{⊤}{\left({\hat{X}}^{*}\right)}^{⊤}{\hat{X}}^{*}\hat{\theta }$$

(24) $$=\left(n_{2}-1\right)\frac{Y^{⊤}Y}{n_{2}-1}-2Y^{⊤}Z^{*}\hat{\beta }\hat{\theta }+{\hat{\theta }}^{⊤}{\hat{\beta }}^{⊤}Z^{*⊤}Z^{*}\hat{\beta }\hat{\theta }$$

(25) $$=\left(n_{2}-1\right)\frac{Y^{⊤}Y}{n_{2}-1}-2n_{2}{\left(\frac{Z^{*⊤}Y}{n_{2}}\right)}^{⊤}\hat{\beta }\hat{\theta }+n_{2}{\hat{\theta }}^{⊤}{\hat{\beta }}^{⊤}\frac{Z^{*⊤}Z^{*}}{n_{2}}\hat{\beta }\hat{\theta }.$$

Observe that $\frac{Y^{⊤}Y}{n_{2}-1}=1$ because we assumed that Y was scaled to have a variance of $1,\frac{Z^{*⊤}Y}{n_{2}}$ can be estimated by $\hat{c},\frac{Z^{*⊤}Z^{*}}{n_{2}}$ can be estimated by $\hat{D}$, and the leftover $n_{2}$ can be replaced by $n^{\prime }$. Therefore, we estimate the RSS by

(26) $$\text{RSS}\approx n^{\prime }\left(1-2{\hat{c}}^{⊤}\hat{\beta }\hat{\theta }+{\hat{\theta }}^{⊤}{\hat{\beta }}^{⊤}\hat{D}\hat{\beta }\hat{\theta }\right)-1.$$

Finally, we estimate the variance of $\hat{\theta }$ by

(27) $$\hat{\text{Var}}(\hat{\theta })={\left({\left({\hat{X}}^{*}\right)}^{⊤}{\hat{X}}^{*}\right)}^{-1}\frac{\text{RSS}}{n_{2}-4}$$

(28) $$\approx {\left({\hat{\beta }}^{⊤}\hat{D}\hat{\beta }\right)}^{-1}\frac{\text{RSS}}{n^{\prime }\left(n^{\prime }-4\right)}.$$

A Wald test for the effect of $\hat{A},$ $\hat{B}$, or $\hat{C}$ on the outcome trait can be performed in the same way as with individual-level data. An F test of overall significance can also be performed using the formula derived for individual-level data, except that the sample size of the individual-level outcome cohort $\left(n_{2}\right)$ should be replaced with the sample size of the GWAS cohort $\left(n^{\prime }\right)$.

### Expression and co-expression imputation models

Any statistical or machine learning algorithms can be used to build expression and co-expression imputation models for COWAS when individual-level data is available for the outcome trait. In order to perform the testing stage of COWAS using summary-level GWAS data, however, the models must be built using an algorithm that can provide a vector of xQTL weights. In this paper, we evaluated penalized linear regression models with three different penalties: the elastic net penalty, the lasso penalty, and the ridge penalty. All models were trained using the glmnet package in R. The α hyperparameter was set to α=0.5 for elastic net regression, α=1 for lasso regression, and α=0 for ridge regression. The λ hyperparameter, which controls the strength of the penalty, was chosen through 10-fold cross validation. Our implementation of COWAS also provides the option for linear regression with stepwise variable selection, but we did not use that method in this study due to its much longer runtime.

To further increase the computational performance of COWAS, we pre-screened genetic variants before including them as features in the penalized regression models. First, we conducted a proteome-wide pQTL mapping study to compute the association between each variant and the standardized residuals of each protein. This enabled us to consider two approaches for pre-screening predictive variants to ensure that only strong pQTLs are considered by each model. For P value screening, we ranked variants by their pQTL P values and kept the top 100. For effect size screening, we instead ranked variants by the absolute values of their pQTL effect sizes and kept the top 100. In both cases, the feature set for the co-expression model was taken to be the union of the top-ranked pQTLs for the two proteins. Note that for the models trained with cis-pQTLs only, we restricted the rankings to variants located close to the gene that codes for the given protein. Performing feature screening before training imputation models greatly decreases the runtime of COWAS, making it computationally feasible to apply to large-scale biobank studies.

It is important to ensure that only well-imputed protein pairs are considered in the testing stage. COWAS assesses the predictive performance of each model by calculating the correlation between imputed and measured expression on a held-out test set. In particular, we randomly selected 80% of the available samples for each protein pair to train imputation models and the remaining 20% to test their predictive performance. For the single-protein models, we calculated the correlation between imputed and measured expression on the test set. Recall that the outcome for the co-expression model, on the other hand, is a quantity estimated using measured expression levels as well as predictions from single-protein models. Thus, to evaluate the performance of the co-expression model, we first obtained $\hat{C}$ using co-expression and single-protein models trained only on the 80% training set. Then we calculated C on the 20% test set, but this time using single-protein models trained on all available data. The out-of-sample correlation for the co-expression model was calculated as the correlation between these estimates of $\hat{C}$ and C. After assessing predictive performance, all three models were re-trained on the full dataset to obtain the final xQTL weight vectors. Only pairs in which all three models had out-of-sample correlations greater than 0.03 were used for hypothesis testing.

### Standard PWAS analysis

We compared our proposed method with the marginal, single-exposure PWAS approach commonly used today. Note that PWAS is independently performed on one protein at a time, so we considered a pair to be significant according to a PWAS analysis if at least one of its proteins was identified by PWAS. Without loss of generality, we will use the notation for protein A to explain the PWAS association test.

Let ${\hat{\beta }}_{A}\in R^{p_{A}}$ be a vector of fitted pQTL weights for imputing the expression of protein A. These weights could be obtained from any regression model, such as the penalized linear regression models we considered in this study. In a setting with individual-level data available for the outcome trait, we first compute

(29) $${\hat{A}}^{*}=Z_{A}^{*}{\hat{\beta }}_{A}$$

where $Z_{A}^{*}$ is a matrix of pQTL genotypes for individuals in the outcome trait dataset. Then we fit the model

(30) $$Y={\hat{A}}^{*}\theta_{A,M}+\varepsilon_{Y,A},$$

where $\theta_{A,M}\in R$ is the coefficient of interest and $\varepsilon_{Y,A}$ is an independent, normally distributed error term. To determine if the genetically regulated component of A has a significant effect on Y, we test the hypothesis $H_{0}:\theta_{A,M}=0$ against its two-sided alternative using a Wald test. The test statistic is ${\left({\hat{\theta }}_{A,M}\right)}^{2}/\text{Var}\left({\hat{\theta }}_{A,M}\right)$, which asymptotically follows a $\chi^{2}$ distribution with 1 degree of freedom under $H_{0}$.

Importantly, note that the PWAS effect size $\theta_{A,M}$ is distinct from the COWAS effect size $\theta_{A}$. Whereas $\theta_{A,M}$ is the marginal effect of $\hat{A}$ on Y, the COWAS coefficient $\theta_{A}$ is the effect of $\hat{A}$ on Y after accounting for the effects of $\hat{B}$ and $\hat{C}$.

In practice, we performed PWAS using summary-level GWAS data for the outcome trait and an LD reference panel. Let ${\hat{c}}_{A}={\left({\hat{c}}_{1},\ldots ,{\hat{c}}_{p_{A}}\right)}^{T}$ be a vector of pseudocor-relations between each of the $p_{A}$ variants in $Z_{A}^{*}$ and the outcome trait Y. Also let ${\hat{D}}_{A}\in R^{p_{A}\times p_{A}}$ be an LD reference panel for those same $p_{A}$ pQTLs. Then we can estimate $\theta_{A,M}$ by

(31) $${\hat{\theta }}_{A,M}={\left({\hat{\beta }}_{A}^{⊤}{\hat{D}}_{A}{\hat{\beta }}_{A}\right)}^{-1}{\hat{\beta }}_{A}^{⊤}{\hat{c}}_{A}.$$

The residual sum of squares for $Y={\hat{A}}^{*}\theta_{A,M}+\varepsilon_{Y,A}$ can be estimated by

(32) $$RSS_{A}\approx n^{\prime }\left(1-2{\hat{c}}_{A}^{⊤}{\hat{\beta }}_{A}{\hat{\theta }}_{A,M}+{\hat{\theta }}_{A,M}^{⊤}{\hat{\beta }}_{A}^{⊤}{\hat{D}}_{A}{\hat{\beta }}_{A}{\hat{\theta }}_{A,M}\right)-1,$$

and finally we can estimate the variance of ${\hat{\theta }}_{A,M}$ by

(33) $$\hat{\text{Var}}\left({\hat{\theta }}_{A,M}\right)={\left({\hat{\beta }}_{A}^{⊤}{\hat{D}}_{A}{\hat{\beta }}_{A}\right)}^{-1}\frac{RSS_{A}}{n^{\prime }\left(n^{\prime }-2\right)},$$

where $n^{\prime }$ is the sample size of the GWAS for Y. Notice that these formulas are analogous to the ones derived for COWAS, except with different degrees of freedom and dimensions for each quantity.

### Data processing and quality control

We trained protein expression and co-expression imputation models on individual-level data from the UKB. First we downloaded genotype data for 92,457,702 autosomal markers and 487,363 samples. Imputation, phasing, and extensive quality control checks had already been performed on the provided data as detailed previously [39]. We further removed any individuals that had a missingness rate greater than 1% across markers, and then subset the data to only keep high-quality samples of genetically-inferred White British ancestry with no relatives of third degree or closer, using indicators provided by UKB. Lastly, we subset the data to individuals who have proteomic data available at the baseline visit. After these steps, 36,171 samples remained.

We also performed additional variant-level quality control on the UKB genotype data. In particular, we removed all variants with a missingness rate greater than 10% across the remaining individuals, those with a minor allele count (MAC) less than 100, those with a minor allele frequency (MAF) less than 1%, and those that failed a Hardy–Weinberg equilibrium test with $P<10^{-15}$. To facilitate matching up variants between UKB data and outcome trait GWAS data, we also removed all variants lacking an rsID and those that are palindromic. Finally, we pruned the variants to $r^{2}<0.8$ with a 1,000 base pair (bp) window and a step size of 100 bp. After these steps, 1,689,714 variants remained. All sample-level and variant-level quality control was done in PLINK 2.00 (https://www.cog-genomics.org/plink/2.0/).

Next, we computed genetic principal components (PCs) from the quality-controlled genotype data. Before computing PCs, we applied several data processing steps in addition to those described in the previous paragraph. In particular, we additionally removed all variants in regions of long-range LD [39] and then pruned the remaining ones to a strict threshold of $r^{2}<0.1$ with a 1,000 bp window and a step size of 100 bp. The computation of genetic PCs was also done in PLINK 2.00.

Proteomic profiling in blood plasma was performed by the UKB Pharma Proteomics Project using the antibody-based Olink Explore 3072 proximity extension assay, which measured 2,941 protein analytes across eight panels and captured 2,923 unique proteins [40]. Various quality control checks and normalization had already been performed as described previously [40]. We downloaded Normalized Protein eXpression (NPX) values for 2,923 proteins in 53,073 samples. Then we subset the data to the set of 36,171 individuals with high-quality genotype data and only kept protein abundance measurements from the baseline visit. The sample sizes for individual proteins ranged from 106 to 35,581 individuals, with a median of 33,643 individuals. Rather than imputing missing values, we used the intersection of samples with non-missing data within each protein pair.

Next, we normalized the NPX levels using a rank-based inverse normal transformation. In particular, we utilized the commonly-used Blom transform with an offset of 3/8 and ties broken by averaging. Following the transformation, we regressed out the following standardized covariates: age, age^2^, sex, age * sex, age^2^ * sex, UKB assessment center, genotyping array, and the first 20 genetic PCs. These protein expression residuals, after normalizing and adjusting for covariates, were used in all downstream analyses.

### Protein annotations

We trained models that include pQTLs screened from across the genome, as well as models that only include *cis*-pQTLs. To identify the *cis*-SNPs for each protein, we obtained start and end positions for the genes coding each assayed protein from annotations provided by the UKB Pharma Proteomics Project [40], and then lifted them over to the hg19 genome build using the UCSC LiftOver web tool (https://genome.ucsc.edu/cgi-bin/hgLiftOver). For proteins coded by several genes, we only considered the first gene listed in the UKB annotation file. We defined the *cis* region for each gene as beginning 500,000 bp upstream of its transcription start site and ending 500,000 bp downstream of its transcription end site. Thus, the *cis*-pQTLs for a given protein are the top-ranked variants that fall within this genomic window.

In this study we only trained models for pairs of proteins found in the HIPPIE database of PPIs [45]. To identify those pairs, we downloaded version 2.3 of the HIPPIE database and mapped each protein in the database to its gene name using the UniProt ID Mapping web tool (https://www.uniprot.org/id-mapping). The gene names were then matched with protein annotations from UKB, and protein pairs present in the HIPPIE database were retained. Note that we considered all protein pairs listed in the HIPPIE database, regardless of their interaction confidence score.

### GWAS data for outcome traits

We considered three complex traits as outcomes in our application of COWAS: low-density lipoprotein (LDL) cholesterol, Alzheimer’s disease (AD), and Parkinson’s disease (PD). The testing stage of COWAS was performed using summary-level data from the largest available GWAS study for each trait. Since none of the GWAS studies provided Z scores in their summary data, we computed them by dividing each variant’s effect size by its standard error.

For LDL cholesterol levels, we downloaded GWAS summary statistics data from the Global Lipids Genetics Consortium (GLGC) [41]. The GLGC aggregated GWAS results from 1,320,016 individuals of European ancestry across 146 cohorts. Their meta-analysis provided summary statistics for 47,006,483 genetic variants and five lipid traits, including LDL cholesterol. Although the authors also conducted a multi-ancestry meta-analysis, we used results that were meta-analyzed solely in the European cohorts to ensure consistency with the genetic ancestry of the majority of UKB participants. A total of 1,624,628 genetic variants remained after harmonization with our quality-controlled UKB genotype data.

For AD status, we downloaded GWAS summary statistics data from the European Alzheimer & Dementia Biobank (EADB) consortium [42]. Namely, we used their stage 1 GWAS of AD and related dementias in individuals of European ancestry. The stage 1 GWAS was a meta-analysis based on 39,106 clinically diagnosed cases, 46,828 proxy cases (with disease status inferred from parental history), and 401,577 controls. Summary statistics for 21,101,114 genetic variants were provided, of which 1,435,986 remained after harmonization with our quality-controlled UKB genotype data.

For PD status, we downloaded GWAS summary statistics data from the International Parkinson Disease Genomics Consortium (IPDGC) [43]. The IPDGC GWAS is also a meta-analysis, aggregating associations across 17 cohorts with individuals of European ancestry. Their main analysis included 37,688 clinically diagnosed cases, 18,618 proxy cases (with disease status inferred from first-degree relatives), and 1,417,791 controls. However, the publicly available summary statistics exclude three studies with individuals from 23andMe due to data sharing restrictions. We used the publicly available GWAS data in our analysis, which was based on 15,056 clinically diagnosed cases, 18,618 proxy cases, and 449,056 controls. Summary-level data was provided for 17,443,094 genetic variants, of which 1,393,959 remained after harmonization with our quality-controlled UKB genotype data.

## Supplementary Material

Supplement 1

Supplement 2

## Data Availability

Genotype, covariate, and protein expression data from the UK Biobank are available through the UK Biobank data access process (https://www.ukbiobank.ac.uk/enable-your-research). Access to the UK Biobank data was approved through UK Biobank Application #35107. Annotations for proteins assayed by the UK Biobank Pharma Proteomics Project are publicly available on Synapse (https://www.synapse.org/Synapse:syn51364943). Protein pairs with known interactions are publicly accessible in the HIPPIE web tool (https://cbdm-01.zdv.uni-mainz.de/~mschaefer/hippie). Publicly available GWAS summary statistics for cholesterol levels were downloaded from the Global Lipids Genetics Consortium website (https://csg.sph.umich.edu/willer/public/glgc-lipids2021). Publicly available GWAS summary statistics for Alzheimer’s disease and Parkinson’s disease were obtained from the NHGRI-EBI GWAS Catalog (https://www.ebi.ac.uk/gwas) under accession numbers GCST90027158 and GCST009325, respectively.
