# Supplementary material for: Co-expression-wide association studies link genetically regulated interactions with complex traits
Source: medRxiv. 2024 Dec 13:2024.10.02.24314813. Preprint. [Version 2] doi: 10.1101/2024.10.02.24314813 (PMC11661334; doi:10.1101/2024.10.02.24314813)
Supplement: Supplementary file 1 [file media-1.pdf]

Supplementary information for "Co-expression-wide association studies link genetically regulated interactions with complex traits"

Mykhaylo M. Malakhov[1] and Wei Pan[1,*]

[1]Division of Biostatistics and Health Data Science, School of Public Health, University of Minnesota, Minneapolis, MN 55414
[*]Correspondence: panxx014@umn.edu

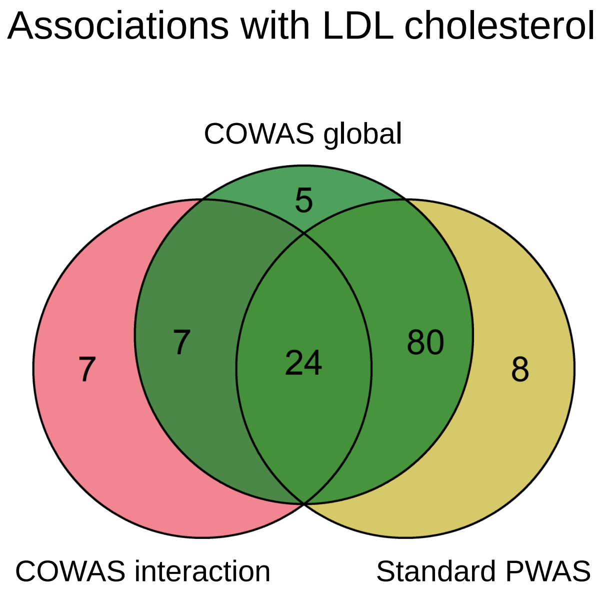

Figure 1: **Numbers of significant protein pairs for LDL cholesterol.**
A Venn diagram displaying the numbers of protein pairs identified as significant for low-density lipoprotein (LDL) cholesterol by the COWAS global test (green), the COWAS interaction test (pink), or a standard PWAS analysis (yellow). Here "standard PWAS" refers to pairs in which at least one of the proteins was identified by PWAS.

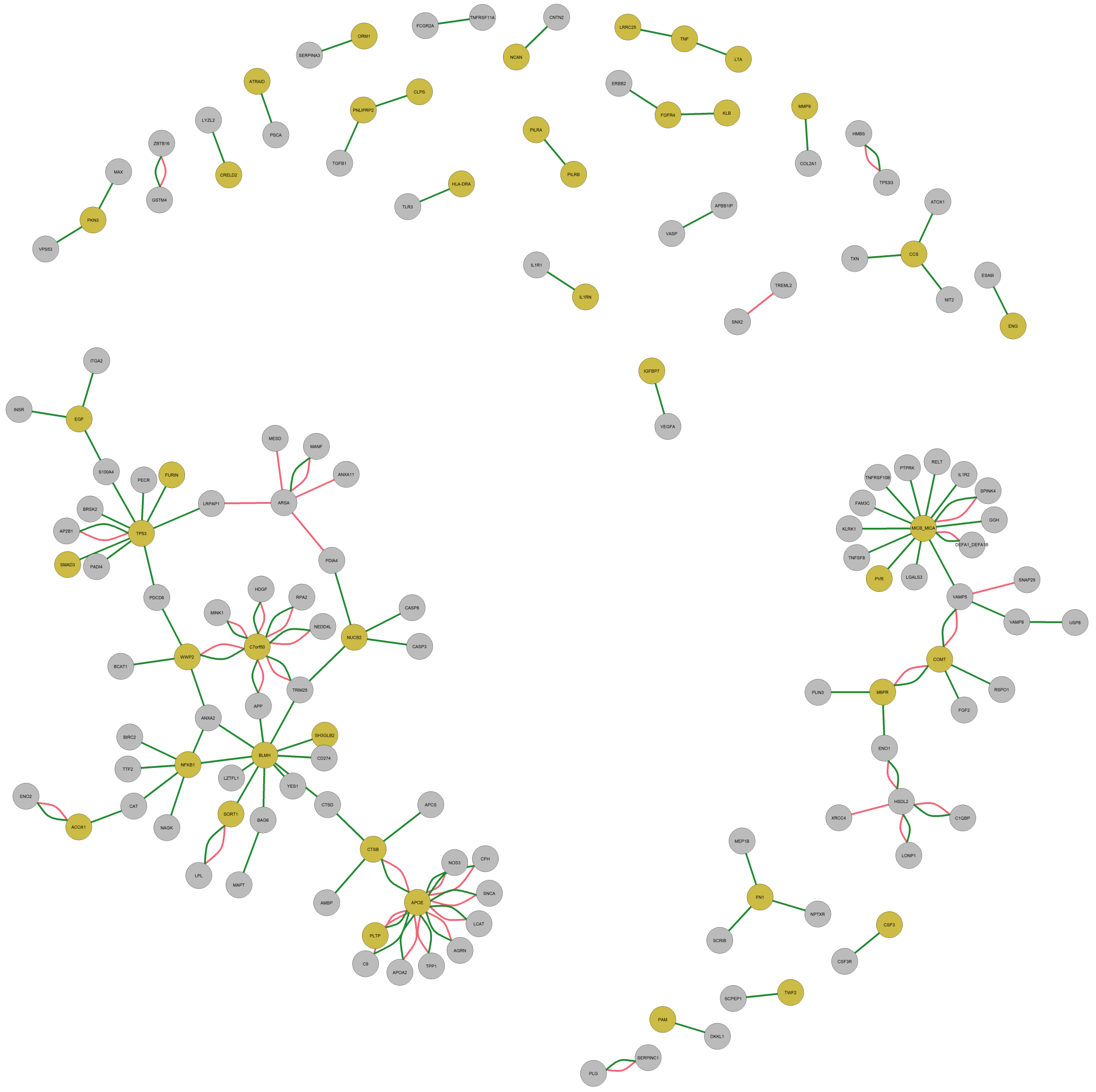

Figure 2: **Network of COWAS discoveries for LDL cholesterol.**
A network diagram showing all of the protein pairs identified as significant for low-density lipoprotein (LDL) cholesterol by either the COWAS global test (green edges) or the COWAS interaction test (pink edges). Node colors indicate whether each protein was identified as significant for LDL by standard PWAS (yellow) or not (gray).

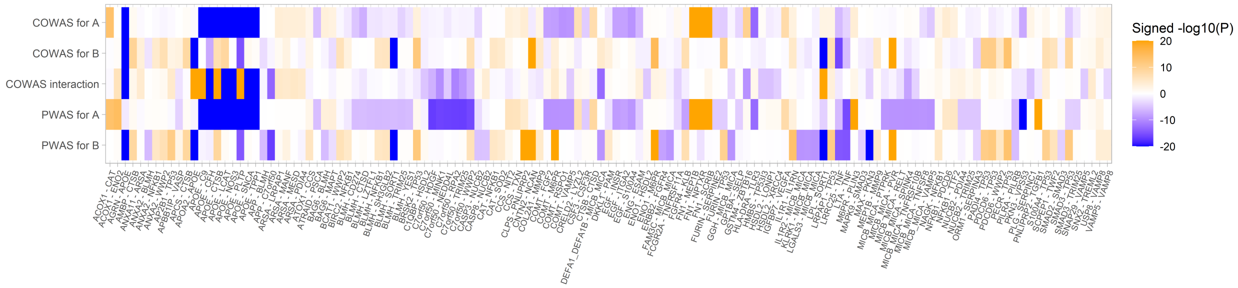

Figure 3: ***P* values and effect directions for LDL cholesterol.**
A heat map displaying signed $-\log_{10}(P)$ values for association with low-density lipoprotein (LDL) cholesterol levels. All protein pairs identified by the COWAS global test, the COWAS interaction test, or a standard PWAS analysis are included in this figure. From top to bottom, the rows correspond to single-protein COWAS tests for proteins A and B, the COWAS interaction test, and PWAS tests for proteins A and B. Here A and B refer to the first and second proteins listed in each pair, respectively. To facilitate visualization, the $-\log_{10}(P)$ values were capped at 20.
